# “Is My Brain Ever Going to Work Fully Again?”: Challenges and Needs of Cancer Survivors with Persistent Cancer-Related Cognitive Impairment

**DOI:** 10.3390/cancers15225331

**Published:** 2023-11-08

**Authors:** Darren Haywood, Evan Dauer, Frank D. Baughman, Blake J. Lawrence, Susan L. Rossell, Nicolas H. Hart, Moira O’Connor

**Affiliations:** 1 Human Performance Research Centre, INSIGHT Research Institute, Faculty of Health, University of Technology Sydney (UTS), Moore Park, NSW 2021, Australia; nicolas.hart@uts.edu.au; 2 Turner Institute for Brain and Mental Health, School of Psychological Sciences, Monash University, Clayton, VIC 3800, Australia; 3 Department of Mental Health, St. Vincent’s Hospital Melbourne, Fitzroy, VIC 3065, Australia; evan.dauer@svha.org.au (E.D.); srossell@srossell.com (S.L.R.); 4 Department of Psychiatry, Faculty of Medicine, Dentistry and Health Sciences, University of Melbourne, Parkville, VIC 3052, Australia; 5 School of Population Health, Curtin University, Bentley, WA 6102, Australia; frank.baughman@curtin.edu.au (F.D.B.); blake.lawrence@curtin.edu.au (B.J.L.); m.oconnor@curtin.edu.au (M.O.); 6 Centre for Mental Health and Brain Sciences, Swinburne University of Technology, Hawthorn, VIC 3122, Australia; 7 Caring Futures Institute, College of Nursing and Health Sciences, Flinders University, Park, SA 5042, Australia; 8 Cancer and Palliative Care Outcomes Centre, Faculty of Health, Queensland University of Technology (QUT), Brisbane City, QLD 4000, Australia; 9 Exercise Medicine Research Institute, School of Medical and Health Science, Edith Cowan University, Joondalup, WA 6027, Australia; 10 Institute for Health Research, University of Notre Dame Australia, Fremantle, WA 6160, Australia

**Keywords:** cancer-related cognitive impairment, chemobrain, qualitative, health professionals, cancer survivors, caregivers, quality of life, needs, challenges

## Abstract

**Simple Summary:**

Many people who have completed cancer treatment experience a ‘fog’ that affects their ability to remember, concentrate, process information, and make decisions. This is known as cancer-related cognitive impairment. There are no purpose-built tools currently available that health professionals can use to understand the needs of cancer survivors who experience cancer related cognitive impairment. To inform the development of such tools, we conducted interviews with (a) cancer survivors with cancer-related cognitive impairment and (b) oncology health professionals to understand the challenges and associated needs of people with cancer-related cognitive impairment. We found five themes describe cancer survivors’ challenges and needs relating to cancer-related cognitive impairment: (1) executing regular activities, (2) relational difficulties, (3) occupational functioning, (4) psychological distress, and (5) social functioning, as well as an additional informational needs domain. This research can be used to inform the development of a purpose-built needs assessment for cancer-related cognitive impairment.

**Abstract:**

Many cancer survivors experience cognitive impairments that impact memory, concentration, speed of information processing, and decision making. These impairments, collectively known as cancer-related cognitive impairments (CRCIs), are a key domain of unmet needs and can significantly impact a cancer survivor’s identity and quality of life. However, there are no purpose-built, multi-domain, needs assessment tools specifically for CRCI. The development of such tools requires an in-depth understanding of cancer survivors’ CRCI-specific challenges and associated needs. This study explored the challenges and associated needs of cancer survivors with persistent CRCI. An in-depth qualitative design using semi-structured interviews with (a) cancer survivors with perceived CRCI (n = 32) and (b) oncology health professionals (n = 19) was utilised. A reflexive thematic analysis of the interviews resulted in five overarching themes: (1) executing regular activities, (2) relational difficulties, (3) occupational functioning, (4) psychological distress, and (5) social functioning, as well as an additional informational needs domain. Ultimately, CRCI was found to directly produce a range of challenges that negatively, and persistently, impact cancer survivors’ quality of life. Cancer survivors were also found to have a range of needs associated with these challenges. This research should be used to inform future challenges and needs assessment tools as well as treatment and supportive care priority areas directly relating to CRCI.

## 1. Introduction

A significant proportion of people who have undergone cancer treatments experience negative impacts on their cognitive ability [1]. Cancer survivors often describe this phenomenon as a ‘fog’ that impacts various cognitive domains, including memory, concentration, speed of information processing, and decision making [1]. This ‘fog’ is commonly referred to as cancer-related cognitive impairment (CRCI) and is estimated to affect between 12% and 75% of cancer survivors [2,3]. CRCI may be present long-term with some cancer survivors experiencing persistent CRCI, defined as the presence of cognitive impairments after cancer treatment completion. People can experience persistent CRCI for up to 20 years. [3].

Although most CRCI research has focused on chemotherapy, CRCI is also prevalent in cancer survivors treated with other therapies, including radiotherapy, immunotherapy, targeted therapy, endocrine therapy, and stem cell transplantation, see [4]. Similarly, CRCI is implicated in a wide range of cancer types, see [1], with CRCI even observed prior to treatment, indicating cancer itself may contribute to CRCI [2]. Recently, Crawford-Williams et al. [5] listed cognitive functioning as one of the key research priorities for cancer survivorship in Australia, and Fan et al. [6] identified CRCI as a key domain of unmet needs.

The causes of CRCI are not fully understood. However, the evidence to date suggests that both biological (i.e., related to the underlying cancer, treatment approaches, and individual patient characteristics) and psychosocial factors may contribute to its development and maintenance. For example, the use of taxane-based chemotherapy is associated with heightened levels of specific cytokines, which can contribute to neurotoxicity, modifications to glial cells, and diminish the capacity for neural repair [7], each likely contributing to CRCI. Further, brain-derived neurotropic factor, which is crucial for regulating neuronal repair and survival as well as synaptic plasticity, is reduced during and after chemotherapy [8]. Psychosocial factors such as depression, anxiety, and general psychological distress are also associated with CRCI [9]. These psychosocial difficulties, which are highly prevalent in cancer survivors, have been theorised to contribute to cognitive slowing and difficulties with sustained attention and decision making; however, this association is not fully understood [10]. Indeed, mental health difficulties are consistently associated with cognitive dysfunction across populations [11,12,13,14,15].

CRCI can have a profound and long-term impact on the individual. Selamat et al.’s [16] review of qualitative CRCI research in breast cancer survivors found that CRCI is real, enduring, and has a major impact on overall quality of life, see also [17]. More recent qualitative evidence with long-term breast cancer survivors suggests CRCI can significantly impact a person’s identity, relationships, and workplace confidence [18]. Further, Lewis and Mackenzie’s [19] scoping review found that women with CRCI returning to work after breast cancer experienced negative impacts on work performance and difficulties with continued participation due to their cognitive changes. It is important to note that there is a lack of qualitative research exploring difficulties associated with CRCI in cancers other than breast cancer, treatments other than chemotherapy, metastatic and advanced cancer, and including genders other than female.

Health professionals show awareness of CRCI and its impacts [20,21]. Multiple biological, cognitive, psychological, and social treatments and supportive approaches aimed to reduce CRCI or support the psychosocial functioning of someone with CRCI have been, and continue to be, developed, see [4,22,23,24]. Mayo et al. [4] describe a range of promising cognitive training, exercise, mind–body, and pharmacological interventions aimed to address CRCI. Occupational support programs have also been developed to facilitate return to work for people with CRCI [25]. While many of these interventions show promise, additional research is required for many of these approaches to be integrated into routine care.

Resources such as the Cancer Council Australia endorsed factsheet, see [20,26], and the overview for oncology providers from the Multinational Association for Supportive Care in Cancer Neurological Complications Study Group [4], provide information regarding best practice of treatment and supportive care options for CRCI based on current evidence. However, even with these resources, health professionals have reported barriers to providing optimal care and support for someone experiencing CRCI, including difficulties with communication regarding CRCI, a lack of specified referral pathways, and a lack of appropriate difficulties and needs assessment tools [20,21]. Health professionals have also reported feeling under-resourced in leading discussions with cancer survivors with CRCI and providing advice due to the uncertainties around CRCI, including the causes, and needs of someone with CRCI, as well as any appropriate referral options [21].

Recent qualitative research with oncology health professionals suggests that a purpose-built needs assessment tool for CRCI [21] would be helpful. While there are validated, purpose-built assessment tools to identify CRCI and understand its impact on functioning and quality of life, see [4,27,28], there are no purpose-built, multi-domain, needs assessment tools specifically for CRCI. Existing needs assessment tools for cancer survivors are typically broad and encompass domains that are often inappropriate for the purpose of exploring the salient needs specifically related to CRCI. For example, measures commonly used in the assessment of the needs of cancer survivors often include domains such as sexual functioning, loss of hair, and tingling and numbness, e.g., [29,30], which may deter health professionals from their use for the specific provision of care for CRCI [21].

Further, items in needs assessments for cancer survivors are typically general (i.e., not related to any particular survivorship challenge, such as CRCI), e.g., [31] and do not elicit the information required to provide optimal care and support for cancer survivors with needs specifically related to their CRCI. A small number of specific assessments exist to explore the cognitive difficulties experienced by survivors of specific cancers, in particular domains of functioning (e.g., the Cognitive Symptom Checklist-Work-59 [32]). However, there is a significant need for a purpose-built needs assessment for persistent CRCI that is relevant across cancer and treatment types and provides information for health professionals to facilitate the optimal choice of treatment, support, and referral options [21].

The development of a widely applicable, purpose-built, needs assessment for persistent CRCI requires an in-depth understanding of CRCI-specific challenges, and associated needs. Therefore, the aim of this study was to explore the challenges and associated needs of cancer survivors with persistent CRCI, specifically relating to their cognitive difficulties, from the perspective of the two primary stakeholder groups: cancer survivors and health professionals.

## 2. Materials and Methods

### 2.1. Design

An in-depth qualitative study design was used to explore the perceived difficulties resulting from CRCI and the associated needs of cancer survivors and health professionals. Further, as this research was grounded within applied settings and was exploratory in nature, we used a constructivist framework within the data collection and analysis. Ethics approval was obtained from St. Vincent’s Hospital Melbourne Human Research Ethics Committee prior to data collection (PID05582) and all actions were conducted in accordance with the Declaration of Helsinki.

### 2.2. Participants and Recruitment

The participants for this study were (1) cancer survivors who had no evidence of disease (any cancer type), had completed any and all treatment for cancer with curative intent, and personally perceived to experience CRCI; and (2) health professionals who work, or have worked, with multiple cancer survivors who completed curative-intent treatment and reported or experienced CRCI. Exclusion criteria for cancer survivors were any specified neurological condition (e.g., traumatic brain injury, dementia, etc.), under the age of 18 years, and could not read and/or converse in English. Exclusion criteria for health professionals were under the age of 18 years and could not read and/or converse in English.

Participants were recruited using a combination of convenience, purposeful, and snowball sampling through social media, health organisations, and researcher networks. Digital flyers with researcher contact information were posted to cancer survivorship and health professional social media groups (with administration permission). Large health organisations such as the Breast Cancer Network Australia’s (BCNA) Review and Survey Group, Bowel Cancer Australia, and Leukaemia Foundation also advertised the study via their networks and social media. The combination of sampling and recruitment approaches allowed for the recruitment of a range of cancer survivors and health professionals, which allowed for the achievement of the study’s aim.

### 2.3. Procedure

After indicating an interest to participate, potential participants received a study information and consent form and were required to provide written consent. Participants then completed a semi-structured interview with a member of the research team. Interviews were conducted face-to-face or online via teleconferencing, and transcribed verbatim. The combination of face-to-face and online interviews were used to facilitate international participant recruitment and to match participant’s preference. The interviews were conducted by the author ED, a registered psychologist and mental health and oncology researcher. ED had prior experience conducting interviews and received additional training and supervision from the research team.

### 2.4. Data Collection Approach and Analysis

Semi-structured interviews, led by two interview guides (one for cancer survivors and one for health professionals) were used for the data collection. Semi-structured interviews are valuable for investigating topics in an exploratory manner since they can be used to gain information that allows for the development of comprehensive insights about the intended subject, while allowing adequate flexibility [33]. To ensure comprehensive data collection, the semi-structured interview guide for cancer survivors was developed informed by the eight key quality of life domains in the Assessment of Quality of Life (AQoL-8D) [34], including independent living, senses, pain, mental health, happiness, self-worth, coping, and relationships. In addition to the eight domains, questions relating to occupational functioning were also included. All questions were phrased in a manner to elicit information directly related to CRCI. Example items include, “What is your experience of being able to cope with life’s problems while having brain fog?”, and “Can you please tell me about your experience of developing and maintaining relationships (i.e., friends, romantic partners, work colleagues) while having brain fog?”. A separate semi-structured interview guide was developed for the health professional interviews. Given the often-restrictive time constraints health professionals have when participating in interviews, we designed a guide with fewer specified items, but allowed for open responses that facilitated rich data collection. Example items included, “What kinds of things does cancer-related cognitive impairment affect?”. Interviews lasted between 30–90 min. Data collection and analysis occurred concurrently, and participant recruitment and data collection continued until information power was reached [35] (i.e., when the necessary depth and richness were obtained that allowed for the achievement of the study aim).

Transcribed data was imported into Quirkos [36] software for use with reflexive thematic analysis [37,38,39]. Reflexive thematic analysis is commonly used within qualitative oncology research, e.g., in [21,40]. Transcripts were read multiple times by ED to facilitate data familiarisation, ED then coded the data by grouping similar meaning units into categories. ED and DH then collaboratively developed the overarching themes. The resultant themes were then refined and finalised through discussion with the entire research team to facilitate reflexivity and multiple perspectives. Recommendations of Pope and Mays [41] were adopted to ensure quality and rigor. An audit trail and reflexive journal were kept detailing both process and personal aspects of the data collection and analysis. Mind maps were also developed and included examples of the justification of why codes and categories were clustered to form a theme. This demonstrated the logical nature of the analytical process that followed the narratives of the participants [42]. These approaches ensured an independent review of the credibility and transferability of the findings [43].

## 3. Results

Overall, 51 people (32 cancer survivors and 19 health professionals) participated in the semi-structured interviews. Cancer survivors were predominantly of breast (56%) or haematological (28%) cancers, ranging from 36–77 years of age (M = 56.03 years, SD = 10.02), and predominantly identified as female (Male = 22%; Female = 78%). Twenty-eight participants resided in Australia, one resided in each of England, South Africa, New Zealand, and the United States. Seven different primary cancer types were represented at varied times since treatment completion. Participant characteristics of cancer survivors are provided in Table 1.

Nineteen oncology health professionals representing thirteen disciplines evenly distributed across the public and private health sectors participated in the study. Health professionals were 31–66 years of age (M = 45.21 years, SD = 10.53), and primarily identified as female (Male = 11%; Female = 89%). Eighteen participants resided in Australia and one in Singapore. Participant characteristics of oncology health professionals are provided in Table 2.

### Qualitative Findings

**Figure 1:**
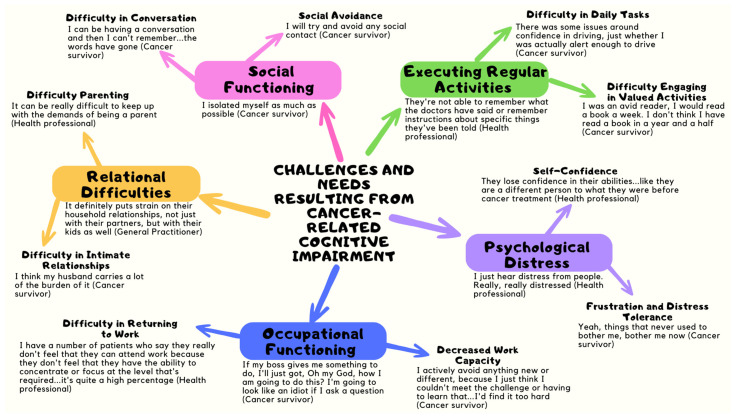
Overarching Themes and Corresponding Sub-Themes.

The findings were organised into five overarching themes: (1) executing regular activities, (2) relational difficulties, (3) occupational functioning, (4) psychological distress, and (5) social functioning. In addition, informational needs related to CRCI were a key element that both groups of participants discussed. Each overarching theme encompasses two sub-themes, discussed in detail in the following sections. Participants’ names have been replaced with pseudonyms. Figure 1 depicts the overarching themes and corresponding subthemes. The supplementary material contains an extended qualitative findings section.

1. Executing Regular Activities

Cancer survivors and health professionals highlighted that CRCI tended to impact one’s capacity to complete regular life activities. Often, they identified common symptoms of memory impairment associated with CRCI that led to difficulty completing tasks such as driving, following a recipe, as well as engaging in valued activities. As such, this theme included the following sub-themes: (a) difficulty in daily tasks and (b) difficulty engaging in valued activities.

  1.1. Difficulties in Daily Tasks

For many cancer survivors, completing daily chores and completing day-to-day tasks was not considered a challenge: *“I’m all good with that stuff” *(Lena). However, this was not the case for all cancer survivors for all activities. Cooking was one such example, with some participants identifying difficulties following recipes while cooking:

*“I remember one day trying to make … pizza dough. We would have made that every week for years and I had the recipe in front of me and it took me an hour to complete it and it should take 5 min …I just couldn’t follow the process”.*

(Freya)

This was echoed by a health professional: *“it takes longer to process the recipe they need to cook dinner” *(Ethan, GP).

Similarly, cancer survivors reported their driving capacity was impaired because of CRCI. At times, this led to reducing how much they drove, or led to increases in having others, typically partners, drive for them. In more severe circumstances, individuals may stop driving altogether, which was the case for one cancer survivor: *“Yeah, I stopped driving, because a few times I had, how did I get here?” *(Matt).

Impaired working memory is a key difficulty for those experiencing CRCI. Forgetfulness was the most reported issue impacting their capacity to execute regular tasks in their lives. Several cancer survivors reported forgetting important items or things around the house. For example, one cancer survivor described repeatedly checking locks in the house due to having many experiences of forgetting to lock or close things:

*“I’ve gone to the drive the car and I thought, I think I closed the door…I don’t think I’ve locked the window or left something on the stove or something. I’ve come back and then I’ve checked and then everything’s done…that happens quite a bit”.*

(Ray)

Another cancer survivor described a situation where she forgot the pin for her card and was unable to pay for her items at the supermarket: *“I was trying to pay for my groceries one day at the supermarket and I just couldn’t remember the pin” (*Maria). Another cancer survivor said: *“Lots of wallets and keys, lots of chasing buses down the street because I left something on it” (Renee).* This participant also engaged in checking behaviour due to her experiences:

*“I plan on the assumption it’s going to happen. I have a very strong habit of checking behind me whenever I get off the bus or leave somewhere…it doesn’t prevent it from happening always”.*

(Renee)

This need to repeatedly check was often due to a lack of confidence in their capacity to remember things. A cancer survivor told a story of needing to pull over his car while driving to a wedding for fear he had forgotten something:

*“We went to a wedding… and I’ve just gone through the list 100 times in my head as I’m driving. Did I do this? Did I do that? Did I pack these? … I had to stop the car and make sure that everything was there”.*

(Ray)

Health professionals also identified how memory issues impacted the capacity of cancer survivors to execute daily tasks. Health professionals tended to focus on how individuals with CRCI often forgot things that were relevant to the work health professionals would do, such as remembering appointments, dates, instructions, and the content of sessions. For example, one health professional reported:

*“They’re not able to remember what the doctors have said or remember instructions about specific things that they’ve been told”.*

(Fiona, Counsellor)

Another said: *“They get frustrated because it’s like…I know that I’m on this medication, been on it for years, but I cannot remember it” *(Maddy, Nurse). Health professionals were also able to identify how it impacted the work they often conducted with their clients, sometimes needing to consider memory issues associated with CRCI. For example, one health professional started a YouTube channel to help their clients remember key concepts during psychological therapy sessions (Lacy, Clinical and Health Psychologist).

  1.2. Difficulty Engaging in Valued Activities

Cancer survivors and health professionals also identified that survivors’ ability to engage in valued behaviours was impaired. The most common valued activity identified was difficulty reading. When working with individuals impacted by CRCI, one health professional said: *“It was the first time I’d seen it in terms of this losing the ability to read” *(Sarah, Nurse). For those participants who enjoyed reading as a hobby, losing this ability to read was a significant loss in their life:

*“I was an avid reader, I would read a book a week. I don’t think I have read a book in a year a half. I read the pages and then I go to bed and then I wake up and I can’t remember the pages I read…”.*

(Stacy)

Another cancer survivor reported that his CRCI impacted his ability to play the guitar:

*“I used to play a lot of guitar… We used to go once a week and play live at a local pub where I used to live and … [had] 60 or 80 songs I remember. These days, if I haven’t got it written in front of me, I can’t get through.*

(Matt)

For another cancer survivor, she described how CRCI meant she could not engage in her hobby of puzzles and crosswords because of associated cognitive difficulties:

*“I used to be really good at crosswords… …when the fog is there, you just can’t do it, you cannot do it”.*

(Reilly)

This is a significant loss in someone’s life and would understandably impact their mood and engagement in life as well as feel a sense of loss for who an individual was before treatment.

Health professionals also recognise that for individuals experiencing CRCI, there is a degree of unmet needs relating to executing daily activities.

*“struggling to navigate, talk about that, you know, can someone else drive you or can you use Google maps…just simple strategies”.*

(Sarah, Nurse)

2. Relational Difficulties

Cancer survivors and health professionals identified an impact on the close relationships of cancer survivors. This often included intimate relationships or an intention to form intimate relationships, a shift in roles within a family unit, the ability to parent their children (for parents with CRCI), as well as CRCI causing frustration and stress for loved ones of those impacted. This theme included the following sub-themes: (a) impacts on intimate relationships and (b) difficulty parenting. One health professional summarised the theme well: *“It definitely…put strains on their household relationships, not just with their partners, but with their kids as well” *(Ethan, GP).

  2.1. Impacts on Intimate Relationships

Cancer survivors and health professionals also identified that CRCI has an impact on individuals as well as the partners of cancer survivors. For many, they reported their daily life had been impacted, causing a change in their role with their intimate partner. For example: *“So she’s taking up a lot of the, I guess things around the house and like just the roles changed like you said” (Matt).* Typically, partners were required to take a more active role in household responsibilities:

*“I would probably ask him to do more things that I normally would do, like I would normally have done all the household things, bill paying and those sorts of things…Now I might say to him can you do that?”.*

(Violet)

This also included daily interactions with partners, with some cancer survivors describing their repetitive apologising for forgetting things. For example:

*“sometimes you have to repeat yourself all the time you kind of go oh, what did you say? What did I say? I can’t remember…I apologise constantly”.*

(Liv)

Furthermore, partners also needed to provide more social and emotional support to cancer survivors. For example, one cancer survivor explained that she struggles with confidence travelling and so required her husband to assist her:

*“He looks after everything and he goes with me everywhere, so I don’t get overwhelmed”.*

(Amelia)

Cancer survivors also recognised this impact on their partners, highlighting they sometimes struggle to cope with the burden: *“I think my husband carries a lot of the burden of it” *(Liv). Health professionals also recognised that partners of those affected by CRCI tend to support them by remembering important things:

*“I think it does pop up in a way that their partners help them to remember things or like is holding those responsibilities to help with that. Or also just remembering to eat, which might sound silly, but I think that does happen quite a lot”.*

(Lily, Dietitian)

For some participants, the impact on their relationship prompted feelings of irritation from their significant other. For example, one cancer survivor said: *“It might take me longer to do it and I can find sometimes my husband might get irritated with me” *(Stacy). Similarly, some cancer survivors reported struggling with their partners because CRCI had altered their identities in some capacity. For example, one cancer survivor said that:

*“it’s unusual because you’re not the person you were when you married”.*

(Amelia)

Another participant expanded on this sentiment, reporting that due to identity change from CRCI he struggles with feeling worthy of his partner:

*“I struggle with, like feelings of not being worthy of her these days…that’s the cause of our friction”.*

These effects were experienced at all stages of relationships: *“I don’t know if people talk to you about the negative impacts on your relationships. I struggled to get relationships and things for many years” *(Sean).

Individuals struggled to retain information about someone they met. For example:

*“It’s hard…making friendships…because I can’t remember a lot about people. It’s not that I don’t care, I care about the person, I just can’t retain any information”.*

(Bella)

  2.2. Difficulty Parenting

For parents, CRCI can also have a significant impact on their capacity to parent their children as well as impact their relationship with their children. Health professionals tended to recognise this more than cancer survivors. Several health professionals noted that they had worked with clients who had a young child or children who they noticed struggling with the ongoing challenges that come with parenting. For example, one health professional said:

*“it can be really difficult to keep up with the demands of being a parent and keeping all of those things that Mum, you know, parents have to keep in their mind when they’re, you know, not on top of their game mentally…it can be very distressing and upsetting” .*

(Nora, Clinical Psychologist)

These difficulties were particularly significant for mothers of young children. One health professional said:

*“We have a number of young mothers and they’re, you know, they’ve got toddlers and they’ve got school age children and they were always able to do everything all at the one time and now it’s just really hard for them to multitask” .*

(Mikayla, Social Worker)

Another health professional had worked with a client who was a new mother where CRCI amplified her difficulties:

*“She’s just devastated, you know, this is worst case scenario, and so for her, she forgets appointments, and she forgets kids’ stuff that you need to carry with you. You know, she’ll take them to a play centre or something and realise that she forgot to take nappies and have to borrow from other parents or turn around and go home” .*

(Emily, Yoga Therapist)

For cancer survivors, they also identified that their relationships with their children were affected, often due to their memory issues. For example, one cancer survivor said:

*“frustration…was unfortunately felt by my daughter”.*

(Ivette)

Health professionals also recognised that individuals affected by CRCI had unmet needs within the theme of relational difficulties. This was mostly around helping to support individuals cope with frustration and stress that may manifest within close relationships:

*“It gives you a bit of a hook to hang your hat on to say…you’re getting really frustrated with your family or frustrated with your kids. That’s something you could be seeing a psychologist about to help with managing frustrations, managing stress”*

(Ethan, GP).

Frustration and irritation between individuals with CRCI and their partners that was reported by cancer survivors and health professionals alike.

3. Occupational Functioning

Both groups described a significant impairment in occupational functioning such as their performance, confidence at work, capacity to work, and new challenges at work because of CRCI. This theme included the following sub-themes: (a) decreased work capacity and (b) difficulty returning to work.

  3.1. Decreased Work Capacity

Cancer survivors reported their capacity to work was reduced compared to pre-cancer function because of CRCI: *“I went back to work and even though it was a job that I did for years and years, it took me a while to pick up the reins again” *(Peter). Often, cancer survivors had difficulty completing tasks they once had no issues completing:

*“I can’t remember how to do this, when I went back to work I couldn’t even remember how to create a Word Document”.*

(Victoria)

Often, cancer survivors reported that tasks needed to be repetitive for them to feel comfortable completing them: *“Automaticity is the key…doing the stuff that you know how to do” *(Renee). Because of this difficulty, cancer survivors reported they would try to avoid challenges and learning new things at work:

*“I actively avoid anything new or different, because I just think I could meet the challenge or having to learn that…I’d find it too hard”.*

(Maria)

Some cancer survivors felt they could not continue to work.

*“I seriously considered resigning from work”.*

(Victoria)

Health professionals also noticed this trend: *“When people feel that they have to quit work because they’re not up to the job anymore or they’re not managing well” *(Fiona, Counsellor). This indicated that CRCI has a significant negative effect on individuals’ confidence at work and, moreover, is likely to lead them to retire earlier, leaving jobs they enjoy for a more ‘straightforward’ job and potentially have a less fulfilling career.

In addition to the day-to-day tasks and requirements of work, cancer survivors also worried about and considered the social component of work. Some reported that working in a team is more challenging because of the social challenges that come with CRCI:

*“It’s harder to work in a team for sure…people I might be working with…would not be very forgiving about me trying to remember something from a couple of minutes ago if I’d forgotten what they’d just told me. It might be perceived as intentional when it really wasn’t”.*

(Ivette)

  3.2. Difficulty Returning to Work

Cancer survivors and health professionals shared similar perspectives on the difficulty of cancer survivors experiencing CRCI to return to work. For most, returning to work is a significant challenge, particularly after completing treatment. One health professional said:

*“I have a number of patients who say they really don’t feel that they can attend work because they don’t feel that they have that ability to concentrate or focus at the level that’s required…it’s quite a high percentage”.*

(Eliza, Nurse)

For cancer survivors, many described difficulties feeling ready to return to work. For example, one said:

*“I’m starting to think that I would, but I’m certainly not ready yet to do it…I wouldn’t feel 100% about being able to manage”.*

(Violet)

Needing to gradually build up their capacity at work was a commonly reported strategy used by cancer survivors: *“So I built up, you know, three days, then four days, then five days” *(Victoria).

Health professionals identified several reasons for individuals struggling to return to work, including anxiety: *“it can heighten the sense of anxiety about this kind of imposter syndrome” *(Tessa, Medical Oncologist), difficulty coping with previous work capacity *“they go back to work and say ‘just can’t cope with what I was doing before’” *(Sarah, Nurse), and impaired cognitive capacity:

*“It has a huge impact on people’s…productivity and ability to work…it really impacts their motivation, focus, and memory”.*

(Mia, Dietitian)

Health professionals had experience of working with individuals who were distressed by their inability to return to work:

*“She had been very academic, very switched on…and she couldn’t return to work and that was really distressing for her”.*

(Emily, Yoga Therapist)

Overall, cancer survivors and health professionals reported that many individuals experiencing CRCI had important unmet needs within the theme of occupational functioning. One cancer survivor said:

*“I just can’t multitask, I can’t, I can’t, and if I’m having a bad day, it’s worse…I’ve got a support worker and hopefully they can help me with getting some work”.*

(Benjamin)

Health professionals also identified the need for individuals experiencing CRCI to be supported in asking for support and assistance while at work so they can better manage their typically impaired work capacity. For example, one health professional said:

*“It’s them feeling really uncomfortable and they need a lot of support with this in asking for adjustments in returning to work”.*

(Lisa, Occupational Therapist)

4. Psychosocial Distress

Cancer survivors and health professionals described CRCI as having significant psychological effects, particularly on their mental health and psychological wellbeing. This theme included the following sub-themes: (a) loss of confidence, and (b) frustration and decreased distress tolerance.

  4.1. Loss of Confidence

A loss of confidence was commonly reported as a psychological difficulty described by cancer survivors and health professionals. Within the psychological distress theme, impaired confidence was the most common issue. One health professional described how her clients struggle to maintain attention at work and as a result have impaired confidence that they can fulfill their duties at work:

*“They suffer as a result of the inability to sustain long periods of attention at work…that really affects the levels of self-confidence”.*

(Robin, Counsellor)

Similarly, a cancer survivor described a lack of confidence to cope in an emergency situation on a boat as he had done in the past:

*“I don’t trust myself to…go driving a boat with passengers these days, because I don’t trust myself to be able to say remember the specific checklist for the engine or to be able to focus in an emergency situation”.*

(Matt)

Cancer survivors also described a loss of confidence in more general terms related to their individual efficacy. One cancer survivor described how her confidence in herself, and her own sense of self has been impaired because of CRCI:

*“It shakes your confidence in yourself, you don’t feel as secure as you were in yourself and certainly, I was pretty comfortable with who I was before, and now I’m not so comfortable”.*

(Chloe)

Health professionals also identified this challenge:

*“They lose confidence in their abilities…like they are a different person to what they were before cancer treatment”.*

(Lisa, Occupational Therapist)

Overall, the loss of a sense of confidence in oneself and one’s capacity to be efficacious in their life had a significant negative impact on those impacted by CRCI and can impede many aspects of functioning.

  4.2. Frustration and Decreased Distress Tolerance

A sense of frustration among cancer survivors affected by persistent CRCI was a very common difficulty reported by cancer survivors and health professionals, due to several reasons. For example, the impact CRCI has on their day-to-day social interactions and capacity to express themselves was commonly reported:

*“I can’t actually find the words for what I wanted to say and then I get really frustrated and I’ll just throw up my hands and walk away”.*

(Chloe)

Health professionals also identified this difficulty, one said: *“there’s some people who get really distressed about it and get really frustrated that they can’t remember things” *(Maddy, Nurse).

Cancer survivors also reported frustration at the sense of loss from ones functioning before they were impacted by CRCI: *“It’s really frustrating because I’m a well-educated person and it’s really frustrating not being able to remember” *(Ivette).

In addition, health professionals noticed that frustration impacted cancer survivors’ daily activities: *“It definitely causes a great deal of frustration and in particular in the daily life, it affects what they can do recreationally” *(Ethan, GP). Being easily bothered was a commonly reported issue associated with the aspect of decreased distress tolerance. One cancer survivor said:

*“Yeah, things that never used to bother me, bother me now… it got really under my skin for some reason…my heart rate went up I ended up going to the doctor”.*

(Ray)

Health professionals identified this risk of reduced distress tolerance and how this may be a risk factor for psychological and emotional issues. One health professional summarised this:

*“I think a lot of time it tends to have a negative effect on mental health”.*

(Ethan, GP)

Within this psychological distress theme, there was a strong emphasis on the unmet need for further information support identified by cancer survivors and health professionals. This centered mostly around needing to provide further psychological and emotional support, typically in the form of access and referral to psychologists. For example, one cancer survivor described how she was concerned and confused about her symptoms of CRCI and sought psychological support:

*“Because I hadn’t been told about it at the beginning…it was quite frightening, you think what the hell is going on? I did actually go and speak to a psychologist”.*

(Ellie)

Health professionals echoed this sentiment, saying:

*“We don’t really think about that but it’s something that comes up all the time with people, but we don’t really explore how distressing it is. But then, you know, that opening the door to saying okay, maybe we need to get a psychologist to…talk to somebody about this”.*

(Maddy, Nurse)

5. Social Functioning

One of the most reported difficulties identified by cancer survivors and health professionals related to CRCI was in the domain of social functioning. The difficulties ranged broadly in this theme, but were considered within two sub-themes: (a) difficulty in conversation and (b) social avoidance.

  5.1. Difficulty in Conversation

Difficulty in conversation was a very common and frustrating difficulty experienced by people with CRCI identified by cancer survivors and health professionals. During interviews, many cancer survivors would lose track of their train of thought: *“Sorry I’ve lost track of where I’m going” *(Matt) or forgot what they were going to say: *“I’m sorry, I’ve forgotten what I wanted to say” *(Violet).

Health professionals also noticed this issue, reporting their clients would often become absent minded when in conversation:

*“Sometimes people just drift off, they just stare off into the distance”.*

(Maddy)

They also recognised the impact this can have on individuals experiencing CRCI, for example, feeling judged in conversations: *“people feel very judged I suppose…they feel like they’re on show” *(Fiona, Counsellor) or silly in front of others: *“they’ll feel really silly in front of their friends and they can find that really confronting” *(Lisa, Occupational Therapist).

For many, they described that they were unable to communicate their thoughts and ideas effectively, sometimes struggling to find words:

*“I think there are sometimes when I think I can feel things, but I can’t find the words very easily”.*

For one cancer survivor, the difficulty in conversation had impacted his feelings of social confidence and identity:

*“I used to be quite assertive, but now I’m not quite as confident in that regard and I can lose track in conversations if there’s two people and uh, like a third or fourth party or a fourth party enters and tries to start another conversation, I’ll completely forget the original conversation”.*

(Sam)

  5.2. Social Avoidance

Many cancer survivors described a tendency to avoid social interactions: *“I will try and avoid any social contact” *(Matt). The reasons for this varied, some cancer survivors reported that they found small talk exhausting and, therefore, avoiding it was preferable:

*“I find it really draining to do it [socially interact], particularly like small talk”.*

(Matt)

Others feared embarrassment in social interactions and thus preferred to avoid them altogether:

*“Having to think of something new and not look like an idiot and then actually having to do it. Not going to happen, I’ll stay at home, it’s easier”.*

(Amelia)

Another cancer survivor reported she is simply not able to engage as well in social interactions and, therefore, it is easier to avoid them: *“It’s just sometimes easier to just not be in social situations than to try and half be in them” *(Renee). Another commonly reported reason for social avoidance was simply a decreased sense of motivation for social interaction: *“I don’t have any motivation to actively seek it out at all” *(Matt).

As a result of these conversational challenges, cancer survivors and health professionals reported that cancer survivors felt they needed to ‘mask’ their CRCI and hide it from others. One health professional said:

*“They tend to hide it, then report it, so when they’re reporting it I feel like they’re quite anxious”.*

(Lacy, Clinical and Health Psychologist)

For cancer survivors, it was described several ways, for example one participant said: *“I’ve worked out a way to camouflage my misunderstanding of stuff” *(Ray). Typically, cancer survivors reported needing to “fake it” more with strangers compared to loved ones: *“with people who are complete strangers, there’s more faking it” *(Renee). Some participants even had certain strategies they used to help to hide their CRCI. For example, cancer survivor said:

*“You learn certain fakey tricks like today, I was saying to this friend, like when you’re losing the word, you stop and meaningfully look up at the sky as though you’re giving great deep though to something, and what you’re doing is thinking of the word”.*

(Renee)

Health professionals also noticed this trend of social avoidance. For example, one health professional said:

*“I think they’re avoiding social situations, a lot of them and that’s bad…you need to have other people in your life. It’s healthy”.*

(Sarah, Nurse)

There was also a preference to be alone or engage in solitary activities by some cancer survivors. For example, one cancer survivor reported: *“I don’t know if it’s because I’ve just, I just get irritated. I don’t want to be around people. I just do my own things…I don’t arrange and do anything anymore” *(Stacy).

For some cancer survivors, this was significant, to the point of withdrawal and isolation: *“I isolated myself as much as possible” *(Sean). This withdrawal included from established relationships:

*“I wouldn’t say I’ve developed any relationships that are more than casual since brain fog and I’ve withdrawn from probably 90% of all the ones that I had”.*

(Matt)

In terms of unmet needs associated with the social functioning theme, cancer survivors and health professionals identified important aspects that would help meet the unmet needs of individuals. This was primarily around the need for psychological support to help to cope and manage the issues that arise from impaired social functioning such as withdrawal and anxiety:

*“The psychologist has helped me to try not put a shell on myself and be more compassionate towards myself…and do things like preparing myself before meeting at a party”.*

(Benjamin)

6. Informational Needs

Cancer survivors and health professionals described a lack of quality information delivered to patients during the treatment process relating to CRCI and the associated need to proactively address this issue. Many cancer survivors described that throughout their treatment journey, they felt they had not received adequate information about the potential for CRCI and duration it may impact them; and in scenarios where they may have been provided information, they often did not retain the information due to the stress of treatment:

*“They give you brochures, but when you’re in that phase of everything, not much of that stuff sinks in”.*

(Sophie)

Health professionals also recognised this issue: *“They get so much information that they can’t possibly retain it all” *(Fiona, Counsellor).

After completing active treatment, there is minimal check-ups or check-ins with health professionals regarding an individual’s recovery:

*“Once you’ve finished your treatment…you don’t really see anybody, there’s no nurse that rings in three months’ time like when you have a baby…they give you a call after six weeks just to check how you’re doing”.*

(Sophie)

Several cancer survivors also identified that they were not informed about CRCI from health professionals during their treatment, and only came across information in online support groups:

*“I don’t remember my medical staff talking about it. I’m in a lot of Facebook support groups and so I do remember hearing about it there in those groups”.*

(Jess)

Some cancer survivors also reported negative experiences if they tried to discuss their CRCI symptoms with health professionals. For example, one cancer survivor said:

*“I had a chat with my haematologist because he asked why I wasn’t back five days a week operational? I said my brain’s fried; I can’t go back operational with a fried brain. He just looked at me as if I had two heads”.*

(Victoria)

Another cancer survivor was told by her oncologist that her symptoms may improve in time but was unable to provide any further information or advice:

*“he said there wasn’t much that he could do about it as there wasn’t much information… it might get better in one to two years and that was kind of basically all I had”.*

(Freya)

For some cancer survivors, there was significant anxiety and fear about when CRCI may last for and its ongoing impact on the functioning of their life. For example, one cancer survivor reported: *“I just get anxious about it and when is it going to stop?” *(Violet). Another cancer survivor reported being concerned about how CRCI may impact her career given she has hopes for a long productive career:

*“I feel like my anxiety around it isn’t much like in the now, but more thinking long term. You know I’m only 39. I have a long career ahead of me still…so I worry that it’s going to continue to decline and impact my work in the future”.*

(Jess)

Health professionals working with cancer survivors and reported that many were worried about their brain function, dementia, or permanent damage. One health professional reported:

*“The worry for them is that you know, is it ever, is my brain ever going to work fully again?”.*

(Mikayla, Social Worker)

Health professionals emphasised the importance of providing information that normalises and validates the cancer survivors’ experiences related to CRCI. For example, one health professional said: *“I would discuss it with patients and talk about, you know, normalising” *(Marlene, Nurse).

In terms of unmet needs associated with CRCI, there was an emphasis by cancer survivors and health professionals on the need for greater information to be provided throughout the cancer journey:

*“When I talked about that, she mentioned chemobrain and she said you know a bit of confusion and what have you…she was quite good just to chat to for a bit of reassurance”.*

(Ellie)

For health professionals, they tended to highlight the need for more information and resources to be provided to cancer survivors and caregivers about CRCI. One health professional reported the importance of helping to provide individuals with relevant information with appropriate language and terminology that can be used to describe CRCI to their friends, family, and workplace:

*“I think really clear information for them…also really, really important to have appropriate terminology that they can take with them with when trying to describe it to their friends or their family or their school or workplace”.*

(Lisa, Occupational Therapist)

Another health professional recognised the need for cancer survivors to be provided with additional information and resources about CRCI: *“the best to do it…it comes with a pamphlet that says, you know, a bit more information, this is resources available” *(Marlene, Nurse) as well as for the carers and loved ones at appointments:

*“This other information is for you and then it can also be used to support the carers or support people who may or may not be at the appointment…even the children and extended family”.*

(Marlene, Nurse)

As pointed out by a health professional, providing appropriate information about CRCI may be protective of mental health difficulties that manifest because of the confusion and distress associated with a lack of understanding of CRCI:

*“I think that, you know, mental health support. Think pre-emptively, actually letting people know what they can expect is helpful because they then they know…then its yes, I was told about this”.*

(Robin, Counsellor)

## 4. Discussion

The aim of this study was to explore the perspectives of cancer survivors and health professionals in order to reveal what challenges and associated needs exist for cancer survivors with persistent CRCI relating to their cognitive difficulties. We identified five overarching themes and ten sub-themes describing cancer survivors’ difficulties and associated needs relating to CRCI were identified: (1) executing regular activities, (2) relational difficulties, (3) occupational functioning, (4) psychological distress, and (5) social functioning. An additional need aspect, namely informational needs, was also prominent among cancer survivors with CRCI.

The performance of tasks and activities in everyday life is a commonly cited difficulty among cancer survivors with CRCI [18]. Our findings mirror and extend upon this understanding by elucidating two specific domains of everyday functioning, namely difficulties completing daily tasks (i.e., cooking and driving), and participation in valued activities (i.e., hobby reading and playing instruments). It is well understood that effective cognitive functioning is fundamental to effective daily functioning, including completing everyday tasks and activities [44]. Activities such as cooking require a range of cognitive processes, including the higher-order processes of executive functioning [45]. For example, updating of working memory is required to effectively maintain relevant information (i.e., required current ingredient amount), while removing previous information from working memory (i.e., the required amount of a previous ingredient), shifting mental set is required to effectively shift attention from one step in the cooking process to the next, and inhibition is required to inhibit a prominent behaviour (i.e., using the same ingredient amounts as usual even if cooking for additional people) [46].

Similarly, many valued activities, such as playing the guitar, place high demands on executive functioning, and many cancer survivors often report difficulties engaging in these types of activities [47]. Health professionals emphasised practical solutions to the unmet needs associated with CRCI’s impact on the performance of daily tasks. Other recent research with health professionals corroborates this finding that advice regarding practical solutions, such as the use of electronic maps when driving, is commonly provided to cancer survivors with CRCI to assist with the performance of everyday tasks and activities [21]. However, regarding the unmet needs relating to difficulties participating in valued activities, such as playing the guitar, practical solutions are not as easily applied [48]. Engagement in cognitively demanding valued behaviours has been suggested to be critical to reducing later-life cognitive decline [49]. Therefore, it is critical that this difficulty and related unmet need is assessed and supportive care is provided. Neuropsychologists and clinical psychologists, due to their understanding of the complex association between cognition and distress related to reduced capability, may be best placed to provide this care.

Cancer survivors with CRCI often encounter relationship difficulties, primarily in intimate and familial relationships, directly resulting from their cognitive difficulties. Intimate relational difficulties have been widely reported in survivorship generally [50,51,52]. However, little has been known about the contribution of CRCI to these difficulties. Henderson et al. [18] have provided initial evidence from their exploratory research with breast cancer survivors that CRCI may have negative impacts on intimate relationships. Our findings extend upon this by demonstrating that cancer survivors, from a range of cancer-types, with persistent CRCI report intimate relationship difficulties resulting directly from their cognitive difficulties. There have been multiple psychosocial interventions developed aimed to improve intimate relationships during cancer survivorship with many of these shown to be effective [52]. However, given the complex multifaceted nature of challenges relating to CRCI, it is fundamental for the intervention facilitator to understand the relational difficulties and needs specifically related to CRCI as experienced by a cancer survivor. This may facilitate mutual communication and understanding between all parties, which is a fundamental aspect of intimate relationship interventions [53], but also is a common challenge relating to CRCI [18,21]. Parenting difficulties were also commonly reported as a challenge for cancer survivors with persistent CRCI. This may be driven by changes in maximal cognitive capacity: the total amount of information one can retain and process [54]. When cognitive capacity is reduced, the multitasking demands associated with parenting may result in the required cognitive load reaching and surpassing current capacity. When the cognitive demand is beyond capacity the required social and other activities cannot be performed to the required level [54], which can result in relational difficulties between parent and child. The assessment and understanding of parental relational difficulties resulting from CRCI is essential for a health professional to offer optimal supportive cancer care, or inform referral practices to an allied health professional, such as a family psychologist or social worker.

Occupational participation and performance after cancer treatment is a growing priority within cancer survivorship and supportive care, e.g., [55,56,57,58,59]. Cancer-related cognitive impairment can have a profound impact on multiple domains of occupational functioning, including returning to work, self-efficacy, and performance. Our findings parallel the growing body of literature that demonstrates that CRCI is a key difficulty and a domain with unmet needs relating to occupational participation and performance [25,60]. Some participants reported they considered resigning, which suggests that CRCI may be a contributing factor accounting for the 26–53% of cancer survivors who quit or lose their jobs within 6 years post-diagnosis [61]. Participants in this study also reported difficulties learning new occupational skills and a preference for repetitive tasks to facilitate automation. This finding parallels the dual-process model of cognition and learning whereby learning a new skill requires a high level of cognitive control supervision, but once highly learned, a skill can become largely autonomous and require little high-order cognitive control or supervision [62]. As CRCI has been shown to affect occupational participation and performance in cancer survivorship, measures have been developed to assess occupational limitations related to CRCI, such as the Cognitive Symptom Checklist-Work [32]. While useful, these measures primarily focus on the cognitive domains contributing to work limitations, with a secondary focus on participation and performance difficulties. For example, the Cognitive Symptom Checklist has three factors: working memory, executive function, and task completion. Further, many of these measures, including the Cognitive Symptom Checklist, are designed for a specific cancer type, such as breast cancer. The assessment of occupational difficulties and needs relating directly to CRCI should be included in future CRCI needs assessments to inform optimal supportive cancer care, treatment approaches, and referral to relevant allied health professionals, including occupational therapists, neuropsychologists, and organisational psychologists.

Psychosocial distress is a commonly experienced challenge for cancer survivors and a key area of supportive care [63,64,65]. The literature shows that psychosocial distress for cancer survivors can be driven by multiple aspects including fear of cancer recurrence, post-traumatic stress, and financial toxicity [66,67,68]. Cancer-related cognitive impairment may be a determining factor of psychosocial distress identified in this study due to its multifaceted impact on identity, efficacy, and capability across multiple domains of life. There is a wide range of supportive care options and interventions, both self-directed and clinician-led, designed to manage psychosocial distress in cancer survivors, see [69]. Specifically regarding CRCI-associated psychosocial distress, two primary aspects of difficulties and related needs require assessment and management, namely (a) self-confidence and (b) frustration and distress tolerance. Self-confidence in a variety of areas can be impacted after cancer [70] and can be driven by real and perceived changes in ability. Cancer survivors’ loss of confidence in their cognitive abilities due to CRCI in this study may be a key driver of psychosocial distress, which extends upon the findings of Matheson [71], that low self-confidence was highly prevalent in men who have had prostate cancer and experience psychological distress, suggesting CRCI may be a key aspect in the association between overall self-confidence and psychosocial distress. Relatedly, the findings of the present study suggest cancer survivors’ changes in cognitive performance may result in a high level of frustration and distress intolerance. Frustration is commonly reported in the qualitative CRCI literature [18,72]. However, the functional relationship between cognitive changes, frustration, and overall psychosocial distress has not been explored. Changes in capabilities resulting from CRCI may lead to a high level of frustration, which in turn contributes to a high level of psychosocial distress. It is, therefore, fundamental to assess and understand the aspects of psychosocial distress directly related to CRCI to provide treatment and supportive options that target these mechanisms of potential action. Future research should also explore this potential functional association.

Social functioning is a key aspect of wellbeing and there has been a focus on exploring and promoting the social functioning of cancer survivors [73,74]. Social functioning refers to a person’s ability to engage in and navigate social interactions and relationships effectively within their context and environment and encompasses the skills and behaviours that facilitate individuals’ communication, cooperation, ability to empathise, and functionally adapt to the expectations and norms [75]. Social functioning is a key domain of quality of life and is suggested to be fundamental to mental and physical wellbeing across the population [34,76,77]. In line with Henderson et al.’s [18] work in breast cancer, the findings of the present study suggest the impacts of cancer and its treatment on cognitive flexibility, memory, and speed of information processing can detrimentally impact a cancer survivor’s ability to communicate effectively. These communication difficulties, as well as changes to confidence and identity, may lead to cancer survivors avoiding social interactions, as identified in this study. This finding is significant as, given the importance of social interaction to cancer survivorship wellbeing, e.g., see [78,79], CRCI’s direct impact on social functioning may result in poorer survivorship outcomes, such as poor mental and/or physical health. Therefore, the importance of the assessment and knowledge gathering relating to the direct impact of CRCI on social functioning is critical to promote wellbeing for cancer survivors.

Cancer survivors’ and caregivers’ unmet needs for additional and higher-quality information are commonly found within the oncology literature [40,80,81]. Indeed, informational needs relating to CRCI have been broadly acknowledged previously [18,20,26]. The findings of the present study broadly support this need for additional information relating to CRCI for cancer survivors. As per Henderson, et al. [18], many participants reported uncertainty relating to the permanency and normality of CRCI and discussed to need for additional information provision from their healthcare team. While participants reported the need for more information, they also reported difficulties in retaining and understanding the wide range of oncology information provided due to their cognitive difficulties. Informational overload is a common issue in cancer care [21,40,82,83], and this may be exacerbated by CRCI. In line with this, easy-to-understand information resources have now been developed that may address the survivors’ needs for additional information while minimising the cognitive load required [20,26]. However, it is suggested that information needs regarding CRCI be assessed prior to information provision to reduce information overload. Therefore, future tools should include the assessment of CRCI-specific informational needs.

### 4.1. Limitations

This study had three primary limitations. First, we did not achieve an even number of participants of each gender, with most participants identifying as female. Whilst this is not uncommon within oncology research generally, and particularly within CRCI research, this does limit confidence in applying our findings more broadly. Secondly, most participants resided in Australia. This may impact the applicability of these findings to others outside of Australia. Lastly, the goal of this study was to explore CRCI difficulties and needs across cancer types and treatments and only within those with persistent CRCI. This has two primary considerations: (1) this sampling limits the findings to general difficulties and needs related to CRCI and does not necessarily capture nuance within cancer type, treatment, or stages; and (2) findings of this study may not be applicable to those currently undergoing cancer treatments.

### 4.2. Directions for Future Research

We intend to use these findings to inform the development of a purpose-built needs assessment for CRCI. This needs assessment will aim to provide information for health professionals to facilitate the optimal choice of treatment, support, and referral options for cancer survivors with persistent CRCI. Beyond this, future research may extend upon our findings by exploring potential differences between cancer types, stages, and treatments regarding the challenges and needs of cancer survivors with persistent CRCI. Further research may also look to further examine challenges and needs related to CRCI within predominantly male samples, as well as samples that are more multinational.

## 5. Conclusions

Ultimately, CRCI is associated with a wide range of challenges that may negatively and persistently impact a cancer survivor’s quality of life. There is also a range of unmet needs directly associated with CRCI-specific challenges, with no existing practical, purpose-built tool to support health professionals in their understanding of the specific needs of cancer survivors. Cancer-related cognitive impairment is found to directly impact cancer survivors’ ability to execute daily activities and tasks, their relationships, their occupational participation and performance, their psychosocial wellbeing, and social functioning. Cancer survivors and health professionals also emphasised the need for additional CRCI-specific information provision. This research should be used to inform future difficulties and needs assessment tools as well as treatment and supportive care priority areas directly relating to CRCI.

## Figures and Tables

**Table 1:** Sample Characteristics for Cancer Survivor Participants.

Characteristic	Mean (SD)/Range/Count
**Age (years)**	56.03 (SD = 10.02, Median = 57.5, Min = 36, Max = 77)
**Gender**	
Male	7
Female	25
**Self-Identified Ethnicity**	
Australian	26
Anglo-Irish	2
New Zealand	1
Maltese/Scottish	1
Anglo-Celtic	1
**Primary Cancer Type**	
Breast	18
Lymphoma	6
Leukemia	3
Bowel	2
Melanoma	1
Ovarian	1
Endometrial	1
**Employment Status**	
Not currently employed	2
Part-Time	9
Full-Time	10
Casual	4
Retired	7
**Education**	1
Secondary School	6
Vocation	13
Bachelor’s degree	9
Master’s Degree	4
**Occupation Sector**	
Healthcare	4
Administration and Reception	3
Transportation	3
Management	3
**Education**	3
Creative work	2
Data analysis	1
Social Services	1
Retail	1
Service professional	1
Police force	1
**When did you first perceive you had ‘brain fog’**	
Before treatment	1
During treatment	17
After treatment	14
**At what stage did you perceive ‘brain fog’ was at its worst?**	
During treatment	6
After treatment	26
Treatments received	
Chemotherapy	29
Radiation	18
Hormone treatment	14
Surgery	18
Stem cell transplant	3
Immunotherapy	1
**Time since completion of active treatment**	5.50 years (SD = 6.09, Median = 2.79 years, Min =1 month, Max = 23 years)

Note. Ethnicity was self-identified through an open response.

**Table 2:** Sample characteristics for health professional participants.

Characteristic	Mean (SD)/Range/Count
**Age (years)**	45.21 (SD = 10.53, Median = 45, Min = 31, Max = 66)
**Gender**	
Male	2
Female	17
Sector	
Public	10
Private	9
**Employment**	
Full-Time	14
Part-Time	5
Education	
Vocation	1
Bachelor’s degree	4
Master’s Degree	11
PhD, MD	3
**Occupation**	
Nurse	5
Counsellor	2
Dietitian	2
Medical Oncologist	1
Radiation Oncologist	1
Clinical Psychologist	1
Health and Clinical Psychologist	1
General Practitioner	1
Occupational Therapist	1
Social Worker	1
Yoga-Therapist	1
Psychologist	1
Exercise Physiologist	1
**Duration in current occupation**	6.88 years (SD = 7.69, Median = 3 years, Min = 11 Months, Max = 25 years)

## Data Availability

De-identified data may be made available by reasonable request of the corresponding author and to the satisfaction of the Human Research Ethics Committee approval.

## Supplementary Materials

The following supporting information can be downloaded at: https://www.mdpi.com/article/10.3390/cancers15225331/s1.
