# Supplementary material for: “Is My Brain Ever Going to Work Fully Again?”: Challenges and Needs of Cancer Survivors with Persistent Cancer-Related Cognitive Impairment
Source: Cancers (Basel). 2023 Nov 8;15(22):5331. doi: 10.3390/cancers15225331 (PMC10669848; doi:10.3390/cancers15225331)
Supplement: Supplementary file 1 [file cancers-15-05331-s001.zip › cancers-2625567-supplementary.pdf]

cancers

MDPI

"Is My Brain Ever Going to Work Fully Again?": Challenges and Needs of Cancer Survivors with Persistent Cancer-Related Cognitive Impairment

Supplementary Material

Extended Qualitative Findings

The findings were organised into 5 overarching themes: (1) executing regular activities, (2) relational difficulties, (3) occupational functioning, (4) psychological distress, and (5) social functioning. In addition, informational needs related to CRCI were a key element that both groups of participants discussed. Each overarching theme encompasses two subthemes, discussed in detail in the following sections. Participants' names have been replaced with pseudonyms. Figure 1 in main depicts the overarching themes and corresponding subthemes.

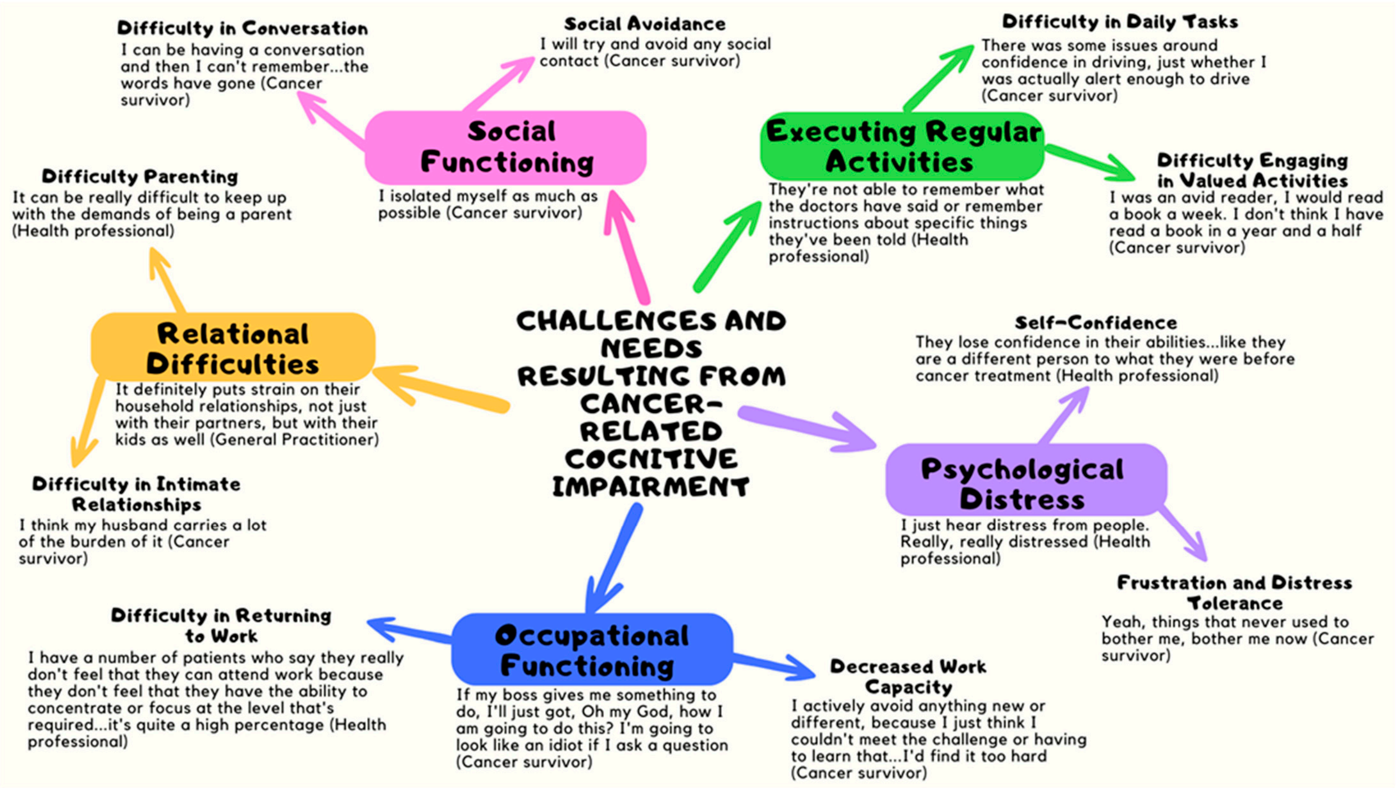

Figure 1 in mian. Overarching Themes and Corresponding Sub-Themes.

1. Executing Regular Activities

Cancer survivors and health professionals highlighted that CRCI tended to impact one's capacity to complete regular life activities. Often, they identified common symptoms of memory impairment associated with CRCI that led to difficulty completing tasks such as driving, following a recipe, as well as engaging in valued activities. As such, this theme included the following sub-themes: (a) difficulty in daily tasks and (b) difficulty engaging in valued activities.

1.1 ***Difficulties in Daily Tasks***

For many cancer survivors, completing daily chores and completing day-to-day tasks was not considered a challenge: *"I'm all good with that stuff" (Lena).* However, this was not the case for all cancer survivors for all activities. Cooking was one such example, with some participants identifying difficulties following recipes while cooking:

"I remember one day trying to make pizza and was making pizza dough. We would have made that every week for years and I had the recipe in front of me and it took me an hour to complete it and it should take 5 minutes and my husband kept coming back in checking…I just couldn't follow the process" (Freya).

This was echoed by a health professional: *"it takes longer to process the recipe they need to cook dinner" (Ethan, GP).*

Similarly, cancer survivors reported their driving capacity was impaired because of CRCI. At times, this led to reducing how much they drove, or led to increases in having others, typically partners, drive for them. One cancer survivor participant reported: *"there was some issues around confidence in driving, just whether I was actually alert enough to drive" (Renee),* with another participant reporting she does not drive long distances anymore to see her family:

"I do find it difficult to concentrate when driving…prior to surgery and treatment I was in the car all the time…my families in Albury and I used to drive down there regularly. There's no way in the world I would attempt to do that now" (Grace). In more severe circumstances, individuals may stop driving altogether, which was the case for one cancer survivor: "Yeah, I stopped driving, because a few times I had, how did I get here?" (Matt).

Impaired working memory is a key difficulty for those experiencing CRCI. Forgetfulness was the most reported issue impacting their capacity to execute regular tasks in their lives. Several cancer survivors reported forgetting important items or things around the house. For example, one cancer survivor described repeatedly checking locks in the house due to having many experiences of forgetting to lock or close things:

"I've gone to the drive the car and I thought, I think I closed the door…I don't think I've locked the window or left something on the stove or something. I've come back and then I've checked and then everything's done…that happens quite a bit" (Ray).

Another cancer survivor described a situation where she forgot the pin for her card and was unable to pay for her items at the supermarket: *"I was trying to pay for my groceries one day at the supermarket and I just couldn't remember the pin" (Maria).* This forgetfulness also meant some individuals had lost items in the past. For example, a cancer survivor said: *"Lots of wallets and keys, lots of chasing buses down the street because I left something on it" (Renee).* This participant also engaged in checking behaviour due to her experiences:

"I plan on the assumption it's going to happen. I have a very strong habit of checking behind me whenever I get off the bus or leave somewhere…it doesn't prevent it from happening always" (Renee).

This need to repeatedly check was often due to a lack of confidence in their capacity to remember things, which again prompted further checking behaviour. A cancer survivor told a story of needing to pull over his car while driving to a wedding for fear he had forgotten something:

"We went to a wedding…it was at the Yarra Valley, so we had to take our clothes up there and everything and I've just gone through the list 100 times in my head as I'm driving. Did I do

this? Did I do that? Did I pack these? And then I confirmed with my wife, I said did I do this? I had to stop the car and make sure that everything was there" (Ray).

Health professionals also identified how memory issues impacted the capacity of cancer survivors to execute daily tasks. Health professionals tended to focus on how individuals with CRCI often forgot things that were relevant to the work health professionals would do, such as remembering appointments, dates, instructions, and the content of sessions. For example, one health professional reported*:*

"They're not able to remember what the doctors have said or remember instructions about specific things that they've been told" (Fiona, Counsellor).

Another said*: "They get frustrated because it's like…I know that I'm on this medication, been on it for years, but I cannot remember it" (Maddy, Nurse).* Health professionals were also able to identify how it impacted the work they often conducted with their clients, sometimes needing to consider memory issues associated with CRCI. For example, one health professional started a YouTube channel to help their clients remember key concepts during psychological therapy sessions (Lacy, Clinical and Health Psychologist).

1.2 *Difficulty Engaging in Valued Activities*

Cancer survivors and health professionals also identified that their ability to engage in valued behaviours was impaired. The most common valued activity identified was difficulty reading. When working with individuals impacted by CRCI, one health professional said: *"It was the first time I'd seen it in terms of this losing the ability to read" (Sarah, Nurse).* For those participants who enjoyed reading as a hobby, losing this ability to read was a significant loss in their life:

"I was an avid reader, I would read a book a week. I don't think I have read a book in a year a half. I read the pages and then I go to bed and then I wake up and I can't remember the pages I read…you don't enjoy it as much because half of it you have forgotten" (Stacy).

Another cancer survivor reported that his CRCI impacted his ability to play the guitar:

"I used to play a lot of guitar… We used to go once a week and play live at a local pub where I used to live and you know, 60 or 80 songs I remember. These days, if I haven't got it written in front of me, I can't get through, I couldn't get through a 3- or 4-minute song without falling apart (Matt).

For another cancer survivor, she described how CRCI meant she could not engage in her hobby of puzzles and crosswords because of associated cognitive difficulties:

"Doing puzzles and things like that, I used to be really good at crosswords…and I find I just look at it and I'll be looking at the clue in the crossword and I would have done it 100 times…when the fog is there, you just can't do it, you cannot do it" (Reilly).

This is a significant loss in someone's life and would understandably impact their mood and engagement in life as well as feel a sense of loss for who an individual was before treatment.

Health professionals also recognise that for individuals experiencing CRCI, there is a degree of unmet needs relating to executing daily activities. Health professionals tended to identify the need for strategies to cope with the day-to-day issues that manifest with CRCI, such as using maps to navigate and using lists and calendars to remember things. For example, one health professional said:

"Then you can help them with strategies to you know, get by…struggling to navigate, talk about that, you know, can someone else drive you or can you use Google maps…just simple strategies (Sarah, Nurse).

2. Relational Difficulties

Cancer survivors and health professionals identified an impact on the close relationships of cancer survivors. This often included intimate relationships or an intention to form intimate relationships, a shift in roles within a family unit, the ability to parent their children (for parents with CRCI), as well as CRCI causing frustration and stress for loved ones of those impacted. This theme included the following sub-themes: (a) impacts on intimate relationships and (b) difficulty parenting. One health professional summarised the theme well: *"It definitely…put strains on their household relationships, not just with their partners, but with their kids as well" (Ethan, GP).*

2.1 ***Impacts on Intimate Relationships***

Cancer survivors and health professionals also identified that CRCI has an impact on individuals as well as the partners of cancer survivors. For many, they reported their daily life had been impacted, causing a change in their role with their intimate partner. For example: *"So she's taking up a lot of the, I guess things around the house and like just the roles changed like you said" (Matt).* Typically, partners were required to take a more active role in household responsibilities:

"I would probably ask him to do more things that I normally would do, like I would normally have done all the household things, bill paying and those sorts of things…Now I might say to him can you do that?" (Violet).

This also included daily interactions with partners, with some cancer survivors describing their repetitive apologising for forgetting things. For example:

"It's actually really hard you know, because sometimes you have to repeat yourself all the time and you don't, you're very conscious of being a batty old lady because you kind of go oh, what did you say? What did I say? I can't remember…I apologise constantly" (Liv).

Furthermore, partners also needed to provide more social and emotional support to cancer survivors. For example, cancer survivor explained that she struggles with confidence travelling and so required her husband to assist her:

"He looks after everything and he goes with me everywhere, so I don't get overwhelmed" (Amelia).

Cancer survivors also recognised this impact on their partners, highlighting they sometimes struggle to cope with the burden: *"I think my husband carries a lot of the burden of it" (Liv).* Health professionals also recognised that partners of those affected by CRCI tend to support them by remembering important things:

"I think it does pop up in a way that their partners help them to remember things or like is holding those responsibilities to help with that. Obviously, a lot of them have a lot of different tablets to take, or also just remembering to eat, which might sound silly, but I think that does happen quite a lot" (Lily, Dietitian).

This often causes frustration for partners and potentially lead to a growing sense of resentment. One health professional said: *"Forgetting things you know, I think if you're already a forgetful person and your partner is remembering everything it's frustrating" (Robin, Counsellor).*

For some participants, the impact on their relationship prompted feelings of irritation from their significant other. For example, one cancer survivor said: *"It might take me longer to do it and I can find sometimes my husband might get irritated with me" (Stacy).* Furthermore, this underlying irritation can often lead to feelings of frustration and conflict, which was also something recognised by cancer survivors. Similarly, some cancer survivors reported struggling with their partners because CRCI had altered their identities in some capacity. For example, one cancer survivor said that:

"it's unusual because you're not the person you were when you married" (Amelia).

Another participant expanded on this sentiment, reporting that due to identity change from CRCI he struggles with feeling worthy of his partner:

"I struggle with, like feelings of not being worthy of her these days…that's the cause of our friction".

Those cancer survivors attempting to form intimate relationships also have significant challenges during the dating process because of CRCI. For example, one cancer survivor emphasised this during the interview: *"I don't know if people talk to you about the negative impacts on your relationships. I struggled to get relationships and things for many years" (Sean).* Another cancer survivor described how she would struggle to recall details of conversations with men she spoke too online before going on a date:

"I've done dating since, but I find it overwhelming getting to know new people...particularly like dating apps…all of a sudden it's this onslaught of people you had to meet and so you go and meet all these different guys, but I couldn't remember things about them…they didn't think I was genuine because I couldn't remember something they told me in our chat…its certainty hard" (Bella).

This was also the case for attempting to form friendships, as the forgetfulness associated with CRCI meant individuals struggled to retain information about someone they met. For example:

"It's hard…making friendships…because I can't remember a lot about people. It's not that I don't care, I care about the person, I just can't retain any information" (Bella).

For some individuals, this is a particularly important difficult as they do not have a long-term spouse from before they were diagnosed with cancer. Meeting and dating partner involves emotional and cognitive functioning as difficult factors for individuals experiencing CRCI and our findings suggest this added load may prompt individuals to avoid attempting to initiate intimate relationships all together.

2.2 Difficulty Parenting

For parents, CRCI can also have a significant impact on their capacity to parent their children as well as impact their relationship with their children. Health professionals tended to recognise this more so compared to cancer survivors. Several health professionals noted that they had worked with clients who had a young child or children who they noticed struggling with the ongoing challenges that come with parenting. For example, one health professional said:

"People who are parents, it can be really difficult to keep up with the demands of being a parent and keeping all of those things that Mum, you know, parents have to keep in their mind when they're, you know, not on top of their game mentally…it can be very distressing and upsetting" (Nora, Clinical Psychologist).

These difficulties were particularly significant for mothers of young children. One health professional said:

"We have a number of young mothers and they're, you know, they've got toddlers and they've got school age children and they were always able to do everything all at the one time and now it's just really hard for them to multitask" (Mikayla, Social Worker).

Another health professional had worked with a client who was a new mother where CRCI amplified her difficulties:

"She's just devastated, you know, this is worst case scenario, and so for her, she forgets appointments, and she forgets kids stuff that you need to carry with you. You know, she'll take them to a play centre or something and realise that she forgot to take nappies and have to borrow from other parents or turn around and go home" (Emily, Yoga Therapist).

For cancer survivors, they also identified that their relationships with their children were impacted, often due to their memory issues. For example, one cancer survivor said:

"Definitely frustration from my daughter when she was younger, because you know, they're not able to forget, understand and forgive as much, especially when they're completely dependent on you to remember things. So, I'd say most frustration…was unfortunately felt by my daughter" (Ivette).

Health professionals also recognised that individuals affected by CRCI had unmet needs within the theme of relational difficulties. This was mostly around helping to support individuals cope with frustration and stress that may manifest within close relationships:

"It gives you a bit of a hook to hang your hat on to say…you're getting really frustrated with your family or frustrated with your kids. That's something you could be seeing a psychologist about to help with managing frustrations, managing stress" (Ethan, GP).

Helping provide individuals and perhaps loved ones with additional psychological and emotional support may aid in reducing the frustration and irritation between individuals with CRCI and their partners that was reported by cancer survivors and health professionals alike.

3. Occupational Functioning

Both groups described a significant impairment in occupational functioning such as their performance, confidence at work, capacity to work, and new challenges at work because of CRCI. This theme included the following sub-themes: (a) decreased work capacity and (b) difficulty returning to work.

3.1 *Decreased Work Capacity*

Cancer survivors reported their capacity to work was reduced compared to pre-cancer function because of CRCI: *"I went back to work and even though it was a job that I did for years and years, it took me a while to pick up the reins again" (Peter).* Oftentimes, cancer survivors had difficulty completing tasks they once had no issues completing:

"I can't remember how to do this, when I went back to work I couldn't even remember how to create a Word Document" (Victoria).

Often, cancer survivors reported that tasks needed to be repetitive for them to feel comfortable completing them: *"Automaticity is the key…doing the stuff that you know how to*

do" (Renee). Because of this difficulty, cancer survivors reported they would try to avoid challenges and learning new things at work:

"I actively avoid anything new or different, because I just think I could meet the challenge or having to learn that…I'd find it too hard" (Maria).

This may have implications for an individual's career development and growth within their organisation or chosen field, thereby impacting their career trajectory.

Some cancer survivors felt they could not continue to work.

"I seriously considered resigned from work" (Victoria).

Health professionals also noticed this trend: *"When people feel that they have to quit work because they're not up to the job anymore or they're not managing well" (Fiona, Counsellor).* This indicated that CRCI has a significant negative effect on individuals' confidence at work and, moreover, is likely to lead them to retire earlier, leaving jobs they enjoy for a more 'straightforward' job and potentially have a less fulfilling career.

In addition to the day-to-day tasks and requirements of work, cancer survivors also worried about and considered the social component of work. Some reported that working in a team is more challenging because of the social challenges that come with CRCI:

"It's harder to work in a team for sure…people I might be working with…would not be very forgiving about me trying to remember something from a couple of minutes ago if I'd forgotten what they'd just told me. It might be perceived as intentional when it really wasn't" (Ivette).

Similarly, there was some fear that asking questions because of impaired memory due to CRCI, may be perceived negatively at work: *"You don't want to be asking questions because then you come across as the one that doesn't understand" (Nick).* This sense of embarrassment was also identified by health professionals: *"Obviously there's a feeling of embarrassment in asking for adjustments, not wanting to be different to their peers at work" (Lisa, Occupational Therapist).*

3.2 Difficulty Returning to Work

Cancer survivors and health professionals shared similar perspectives on the difficulty of cancer survivors experiencing CRCI to return to work. For most, returning to work is a significant challenge, particularly after completing treatment. One health professional said:

"I have a number of patients who say they really don't feel that they can attend work because they don't feel that they have that ability to concentrate or focus at the level that's required…it's quite a high percentage" (Eliza, Nurse).

For cancer survivors, many described difficulties feeling ready to return to work. For example, one said:

"I'm starting to think that I would, but I'm certainly not ready yet to do it…I wouldn't feel 100% about being able to manage" (Violet).

Needing to gradually build up their capacity at work was a commonly reported strategy used by cancer survivors: *"So I built up, you know, three days, then four days, then five days" (Victoria).* Evidently, this suggests that cancer survivors have significant barriers to returning to work, particularly feeling psychologically ready to return and feeling they can not cope. This may delay re-engaging in an important and meaningful component of

life. On a more practical level, the financial pressure and burden for cancer survivors to support themselves is exacerbated when CRCI impacts returning to work.

Health professionals had similar perspectives, noting that:

"they can't work…in their roles as they normally would" (Mikayla, Social Worker).

Health professionals identified several reasons for individuals struggling to return to work, including anxiety: *"it can heighten the sense of anxiety about this kind of imposter syndrome" (Tessa, Medical Oncologist),* difficulty coping with previous work capacity *"they go back to work and just can't cope with what I was doing before" (Sarah, Nurse),* and impaired cognitive capacity:

"It has a huge impact on peoples…productivity and ability to work…it really impacts their motivation, focus, and memory" (Mia, Dietitian).

Therefore, from the perspective of health professionals, there is a wide range of difficulties for those returning to work with CRCI, highlighting the barriers that prevent individuals from returning to work.

Difficulty returning to work significantly impacted the lives of cancer survivors, often causing distress. Health professionals had experience of working with individuals who were distressed by their inability to return to work:

"She had been very academic, very switched on…and she couldn't return to work and that was really distressing for her" (Emily, Yoga Therapist).

Overall, cancer survivors and health professionals reported that many individuals experiencing CRCI had important unmet needs within the theme of occupational functioning. One cancer survivor said:

"I just can't multitask, I can't, I can't, and if I'm having a bad day, it's worse…I've got a support worker and hopefully they can help me with getting some work" (Benjamin).

This highlights that for those impacted by CRCI without work, having practical assistance and support in securing work is significantly important. Providing this support would ensure the ideal implementation of cancer treatment support for individuals. Health professionals also identified the need for individuals experiencing CRCI to be supported in asking for support and assistance while at work so they can better manage their typically impaired work capacity. For example, one health professional said:

"It's them feeling really uncomfortable and they need a lot of support with this in asking for adjustments in returning to work" (Lisa, Occupational Therapist).

Being able to provide this support will ensure individuals are more comfortable at work and may increase the likelihood they return and remain at work after a difficult period away due to treatment.

4. Psychosocial Distress

Cancer survivors and health professionals described CRCI as having significant psychological effects, particularly on their mental health and psychological wellbeing. This theme included the following sub-themes: (a) loss of confidence, and (b) frustration and decreased distress tolerance.

4.1 Loss of Confidence

A loss of confidence was commonly reported as a psychological difficulty described by cancer survivors and health professionals. Within the psychological distress theme, impaired confidence was the most common issue: *"I would say confidence is the biggest one though" (Fiona, Counsellor).* This loss of confidence is typically related to losing the sense of efficacy to do what they once did in life. For example, one health professional described how her clients struggle to maintain attention at work and as a result have impaired confidence that they can fulfill their duties at work:

"They suffer as a result of the inability to sustain long periods of attention at work…that really affects the levels of self-confidence" (Robin, Counsellor).

Similarly, a cancer survivor described a lack of confidence to cope in an emergency situation on a boat as he had done in the past:

"I don't trust myself to…go driving a boat with passengers these days, because I don't trust myself to be able to say remember the specific checklist for the engine or to be able to focus in an emergency situation" (Matt).

Cancer survivors also described a loss of confidence in more general terms related to their individual efficacy. One cancer survivor described how her confidence in herself, and her own sense of self has been impaired because of CRCI:

"It shakes your confidence in yourself, you don't feel as secure as you were in yourself and certainly, I was pretty comfortable with who I was before, and now I'm not so comfortable" (Chloe).

This was echoed by another cancer survivor: *"I'd say your self-confidence does take a bit of a blow and you're now as assured of yourself as what you were before" (Stacy).* Health professionals also identified this challenge:

"They lose confidence in their abilities…like they are a different person to what they were before cancer treatment" (Lisa, Occupational Therapist).

Overall, the loss of a sense of confidence in oneself and one's capacity to be efficacious in their life had a significant negative impact on those impacted by CRCI and can impede many aspects of functioning.

4.2 Frustration and Decreased Distress Tolerance

A sense of frustration among cancer survivors affected by persistent CRCI was a very common difficulty reported by cancer survivors and health professionals, due to several reasons. For example, the impact CRCI has on their day-to-day social interactions and capacity to express themselves was commonly reported:

"I can't actually find the words for what I wanted to say and then I get really frustrated and I'll just throw up my hands and walk away" (Chloe).

Similarly, being forgetful in conversation led to a lot of frustration for cancer survivors: *"frustration with, you know, I have to apologise a lot to people because I'm sorry I've forgotten" (Chloe).* Another cancer survivor, a teacher, found it frustrating that she often forgot her student's names:

"It's frustrating, it's upsetting that, you know, you've got these gorgeous kids that you know, Why can't I remember their name? Why isn't it coming to me?" (Raffaela).

Health professionals also identified this difficulty, one said: *"there's some people who get really distressed about it and get really frustrated that they can't remember things" (Maddy, Nurse).*

Cancer survivors also reported frustration at the sense of loss from ones functioning before they were impacted by CRCI: *"It's really frustrating because I'm a well-educated person and it's really frustrating not being able to remember" (Ivette).* Another said:

"I get frustrated by the fact that I used to have this laser focus and be very efficient at things and that's just wiped completely" (Matt).

This sense of loss of their former self was a commonly reported difficulty when experiencing CRCI, with the most reported impact being a strong feeling of frustration.

Health professionals also identified these issues, highlighting significant frustration with social interactions by cancer survivors which impacted their relationships with significant others:

"Frustration and anger creeps into conversations, especially with loved ones" (Maddy, Nurse).

In addition, health professionals noticed that frustration impacted cancer survivors' daily activities: *"It definitely causes a great deal of frustration and in particular in the daily life, it affects what they can do recreationally" (Ethan, GP).* One health professional gave an example of a patient she knew who had lost the capacity to play a strategy game because of CRCI which caused her frustration:

"She kind of talked about it quite a lot that you know you have to give signals…it's strategic, it's strategy game I guess she's not good at it anymore or not as good as she was and so that's frustrating that she feels she can't play" (Emily, Yoga Therapist).

Participants also identified that, for individuals experiencing CRCI, there was often a reduction in their capacity to cope with distress, where they were more easily bothered by things or had more intense emotional reactions to events compared to what they had in the past. One cancer survivor highlighted that at times, she simply becomes highly distressed and cries for no apparent reason:

"You become very emotional, I mean on Tuesday I was in tears for no reason. My husband walked in, I just started crying and he said "You're okay" and I was like, well, fine." (Stacy).

This emotional volatility was relatively common among cancer survivors and indicates that CRCI seemingly impacts their emotional state, and often tips individuals into feeling overwhelmed. Moreover, as demonstrated in this quote, partners and spouses need to provide additional emotional support because of decreased emotional tolerance associated with CRCI among cancer survivors.

Being easily bothered was a commonly reported issue associated with the aspect of decreased distress tolerance. One cancer survivor said:

"Yeah, things that never used to bother me, bother me now…I had applied for an internal job and someone else was chosen…I wasn't even interviewed and that was so frustrating, but before it would have been like water off a ducks back…it got really under my skin for some reason…my heart rate went up I ended up going to the doctor" (Ray).

This highlights how a stressor is experienced as significantly more distressing and upsetting compared to previous functioning before their CRCI. Psychologically and emotionally, this is a factor that may tilt an individual to experience life's events as more

negative and distressing and, in severe cases, may be a predisposing factor to mental health issues, including depression and anxiety. Health professionals identified this risk of reduced distress tolerance and how this may be a risk factor for psychological and emotional issues. One health professional summarised this:

"I think a lot of time it tends to have a negative effect on mental health" (Ethan, GP).

Within this psychological distress theme, there was a strong emphasis on the unmet need for further information support identified by cancer survivors and health professionals. This centred mostly around needing to provide further psychological and emotional support, typically in the form of access and referral to psychologists. For example, one cancer survivor described how she was concerned and confused about her symptoms of CRCI and sought psychological support:

"Because I hadn't been told about it at the beginning…it was quite frightening, you think what the hell is going on? I did actually go and speak to a psychologist" (Ellie).

Another cancer survivor felt strongly that counselling should be available to everyone completing cancer treatment due to CRCI symptoms:

"I think you should get six rounds of counselling afterwards, its not mandatory…but with the chemobrain I think it would help give other coping mechanisms (Victoria)".

Health professionals echoed this sentiment, saying:

"We don't really think about that but it's something that comes up all the time with people, but we don't really explore how distressing it is. But then, you know, that opening the door to saying okay, maybe we need to get a psychologist to…talk to somebody about this" (Maddy, Nurse).

Another health professional said: *"psychology to discuss any stressors or concerns that they may have and how to best manage those symptoms (Meredith, Exercise Physiologist).* In more severe cases where clients or patients are struggling significantly with mental health issues, health professionals identified that psychiatric intervention from psychiatrists may be necessary. For example: *"Sometimes they might, see a psychiatrist if they're quite depressed, so they might need some help with that" (Sean, Nurse)*

5. Social Functioning

One of the most reported difficulties identified by cancer survivors and health professionals related to CRCI was in the domain of social functioning. The difficulties ranged broadly in this theme but were considered within two sub-themes: (a) difficulty in conversation and (b) social avoidance.

5.1 Difficulty in Conversation

Difficulty in conversation was a very common and frustrating difficulty experienced by people with CRCI identified by cancer survivors and health professionals. This subtheme was also directly observed by our research team when conducting semi-structured interviews. During the interviews, many cancer survivors would lose track of their train of thought: *"Sorry I've lost track of where I'm going" (Matt)* or forgot what they were going to say: *"I'm sorry, I've forgotten what I wanted to say" (Violet*). For many of the interviews, participants would ask for the question to be repeated:

"I keep forgetting what you just asked me and that would never have happened before…say, what was the question?" (Ivette).

Although this was not a significant issue during the interview process, this social functioning difficulty was highly apparent. Day-to-day interactions may require increased effort and concentration, and individuals may feel embarrassed that they have forgotten someone's question, leading to feelings of embarrassment and awkwardness in these interactions.

Health professionals also noticed this issue, reporting their clients would often become absent minded when in conversation:

"Sometimes people just drift off, they just stare off into the distance" (Maddy).

They also recognised the impact this can have on individuals experiencing CRCI, for example, feeling judged in conversations: *"people feel very judged I suppose…they feel like they're on show" (Fiona, Counsellor)* or silly in front of others: *"they'll feel really silly in front of their friends and they can find that really confronting" (Lisa, Occupational Therapist).*

For cancer survivors, the difficulty in conversation arose for several reported reasons. One of the most common reasons was forgetfulness in conversation:

"I can be having a conversation and then I can't remember…the words have gone" (Violet).

For many, they described that they were unable to communicate their thoughts and ideas effectively, sometimes struggling to find words:

"I think there are sometimes when I think I can feel things, but I can't find the words very easily".

For some, this difficulty with communicating their thoughts and ideas can impede their ability communicate in tense situations. For example, one cancer survivor described a situation where he wanted to say something at his Godson's rugby game to defend his team but was unable too:

"I went to watch a rugby match yesterday and my Godson was playing. There were young student lads, rugby types on the sideline throwing abuse at his team and I really wanted to say something. I had something prepared in my head and I repeated it over and over and I thought this will be good, this will be witty, and when it came out, it didn't come out in the order…it was different to how I wanted it to come out" (Sean).

Difficulty in conversation lead to significant consequences for some cancer survivors. For one cancer survivor, the difficulty in conversation had impacted his feelings of social confidence and identity:

"I used to be quite assertive, but now I'm not quite as confident in that regard and I can lose track in conversations if there's two people and uh, like a third or fourth party or a fourth party enters and tries to start another conversation, I'll completely forget the original conversation" (Sam).

5.2 Social Avoidance

Many cancer survivors described a tendency to avoid social interactions: *"I will try and avoid any social contact" (Matt).* The reasons for this varied, some cancer survivors reported that they found small talk exhausting and therefore avoiding it was preferable:

"I find it really draining to do it [socially interact], particularly like small talk" (Matt).

Others feared embarrassment in social interactions and thus preferred to avoid them altogether:

"Having to think of something new and not look like an idiot and then actually having to do it. Not going to happen, I'll stay at home, it's easier" (Amelia).

Another cancer survivor reported she is simply not able to engage as well in social interacts and therefore it is easier to avoid them: *"It's just sometimes easier to just not be in social situations than to try and half be in them" (Renee).* Another commonly reported reason for social avoidance was simply a decreased sense of motivation for social interaction*: "I don't have any motivation to actively seek it out at all" (Matt).*

As a result of these conversational challenges, cancer survivors and health professionals reported that cancer survivors felt they needed to 'mask' their CRCI and hide it from others. One health professional said:

"They tend to hide it, then report it, so when they're reporting it I feel like they're quite anxious" (Lacy, Clinical and Health Psychologist).

For cancer survivors, it was described several ways, for example one participant said: *"I've worked out a way to camouflage my misunderstanding of stuff" (Ray),* while another said: *"I am pretty adept at trying to hide it" (Chloe).* Typically, cancer survivors reported needing to "fake it" more with strangers compared to loved ones: *"with people who are complete strangers, there's more faking it" (Renee).* Some participants even had certain strategies they utilised to help to hide their CRCI. For example, cancer survivor said:

"You learn certain fakey tricks like today, I was saying to this friend, like when you're losing the word, you stop and meaningfully look up at the sky as though you're giving great deep though to something, and what you're doing is thinking of the word" (Renee).

Another strategy was to turn any social forgetfulness into a joke:

"If I make a mistake, I'll try and convert it into a joke…someone will say to me, I'll ask them something and they'll say "we did it last week, don't you remember?" …look I'm just testing you know, I kind of play games like that" (Ray).

Health professionals also noticed this trend of social avoidance. For example, one health professional said:

"I think they're avoiding social situations, a lot of them and that's bad…you need to have other people in your life. It's healthy" (Sarah, Nurse).

Another reported that: *"they might withdraw from social activities" (Lisa, Occupational Therapist).* Generally, the feeling of need to mask or hide their difficulty in conversation was the result of wanting to portray a sense of being in control. However, there was often a sense that others did not understand the individual's difficulties and would view them negatively for their forgetfulness or difficulty following conversation.

There was also a sense of preferring to be alone or engage in solitary activities by some cancer survivors. For example, one cancer survivor reported that she preferred not to be around people as she was easily irritated by social interactions with others:

"I don't know if it's because I've just, I just get irritated. I don't want to be around people. I just do my own things…I don't arrange and do anything anymore" (Stacy).

For some cancer survivors, this was more significant, with a strong desire to not engage with others to the point of withdrawal and isolation: *"I isolated myself as much as possible" (Sean).* This withdrawal included from established relationships:

"I wouldn't say I've developed any relationships that are more than casual since brain fog and I've withdrawn from probably 90% of all the ones that I had" (Matt).

Similarly, another cancer survivor reported that she has been lying to avoid seeing others:

"I found myself saying to myself I just wish people would leave me alone, it's just all too overwhelming…I find myself telling little white lies once I've made plans" (Julia).

This demonstrates the extent of the significant negative impact CRCI can have on an individual's social functioning. The described issues and subsequent withdrawal for some will have significant implications for the quality of their relationships and mental health, potentially putting them at increased risk for a whole range of mental health issues including depression and anxiety as well as decreased quality of life.

In terms of needs associated with the social functioning theme, cancer survivors and health professionals identified important aspects that would help meet the unmet needs of individuals. These supports looked different for each group of participants. For health professionals, some suggested referring clients to speech pathologists to assist with word-finding difficulties that occur in social interactions. For example:

"Potentially a referral...if it's word finding, a speech pathologist" (Meredith, Exercise Physiologist).

In contrast, cancer survivors reported feeling the need for psychological support to help to cope and manage the issues that arise from impaired social functioning such as withdrawal and anxiety:

"The psychologist has helped me to try not put a shell on myself and be more compassionate towards myself…and do things like preparing myself before meeting at a party" (Benjamin).

Addressing these unmet needs identified would assist individuals in their social interactions at work, with friends, and loved ones and prevent the potential negative side effects outlined such as withdrawal and engaging in solitary activities as a form of avoidance.

6. Informational Needs

Cancer survivors and health professionals described a lack of quality information delivered to patients during the treatment process relating to CRCI and the associated need to proactively address this issue. Many cancer survivors described that throughout their treatment journey, they felt they had not received adequate information about the potential for CRCI and duration it may impact them; and in scenarios where they may have been provided information, they often did not retain the information due to the stress of treatment:

"They give you brochures, but when you're in that phase of everything, not much of that stuff sinks in" (Sophie).

This was echoed by another cancer survivor: *"I can't remember being told until after I started having the problem I think, but I can't be 100% sure" (Isabella).* Health professionals also recognised this issue: *"They get so much information that they can't possibly retain it all" (Fiona, Counsellor).*

Furthermore, it was identified that after completing active treatment, there is minimal check-ups or check-ins with health professionals regarding an individual's recovery:

"Once you've finished your treatment…you don't really see anybody, there's no nurse that rings in three months' time like when you have a baby…they give you a call after six weeks just to check how you're doing" (Sophie).

Given that CRCI can last significantly longer than 6 weeks for many individuals, a lack of ongoing long-term check in's is an issue for delivering optimal treatment which considers psychological, emotional, physical, and social functioning.

Several cancer survivors also identified that they were not informed about CRCI from health professionals during their treatment, and only came across information in online support groups:

"I don't remember my medical staff talking about it. I'm in a lot of Facebook support groups and so I do remember hearing about it there in those groups" (Jess).

Some cancer survivors also reported negative experiences if they tried to discuss their CRCI symptoms with health professionals. For example, one cancer survivor said:

"I had a chat with my haematologist because he asked why I wasn't back five days a week operational? I said my brain's fried; I can't go back operational with a fried brain. He just looked at me as if I had two heads" (Victoria).

Another cancer survivor was told by her oncologist that her symptoms may improve in time but was unable to provide any further information or advice:

"I know my oncologist mentioned it…he said there wasn't much that he could do about it as there wasn't much information. It was just like, you know, it might get better in one to two years and that was kind of basically all I had" (Freya).

This is a concern, as for some cancer survivors, there was significant anxiety and fear about when CRCI may last for and its ongoing impact on the functioning of their life. For example, one cancer survivor reported: *"I just get anxious about it and when is it going to stop?" (Violet).* Another cancer survivor reported being concerned about how CRCI may impact her career given she has hopes for a long productive career:

"I feel like my anxiety around it isn't much like in the now, but more thinking long term. You know I'm only 39. I have a long career ahead of me still…so I worry that it's going to continue to decline and impact my work in the future" (Jess).

Moreover, some cancer survivors questioned their sanity or if they were developing dementia due to lack of information about CRCI:

"The doctors certainly weren't interested…I think there is a real big gap there and it should be addressed because you feel sometimes like you are going crazy" (Grace).

This was also echoed by health professionals, many of whom had experience working with cancer survivors who worried about their brain function, dementia, or permanent damage. One health professional reported:

"The worry for them is that you know, is it ever, is my brain ever going to work fully again?" (Mikayla, Social Worker).

Health professionals emphasised the importance of normalising and validating the cancer survivors' experiences related to CRCI. For example, one health professional said: *"I would discuss it with patients and talk about, you know, normalising" (Marlene, Nurse).* Typically, normalising involved informing cancer survivors they are not alone in their

experience of CRCI and that it is a common side effect of cancer and cancer treatment. For example, one health professional reported:

"I will tell them generally that it is actually really common with the therapy that they've received or the therapy that they're on" (Tessa, Medical Oncologist).

In terms of needs associated with CRCI, there was an emphasis by cancer survivors and health professionals on the need for greater information to be provided throughout the cancer journey. For cancer survivors, they identified that greater information about CRCI being provided during their cancer treatment journey would have been helpful and reassured them the issues they were having were a common side effect. For example, one cancer survivor identified that her psychologist was very helpful in reassuring her about the symptoms of CRCI:

"When I talked about that, she mentioned chemobrain and she said you know a bit of confusion and what have you…she was quite good just to chat to for a bit of reassurance" (Ellie).

Another cancer survivor reported that she struggled to find any professional help or support and eventually had to turn to doing her own research to understand CRCI:

"I just felt that nobody wanted to listen…I have since had to find a new Doctor because all the times that I needed him, I couldn't get in to see him…I didn't have access to the breast care nurses…So I just gave up, oh well I'm researching and dealing with it all myself" (Grace).

This highlights the significant need for health professionals to have a comprehensive understanding of CRCI to be able to identify, validate, normalise, and provide support or referrals for patients experiencing CRCI.

For health professionals, they tended to highlight the need for more information and resources to be provided to cancer survivors and caregivers about CRCI. One health professional reported the importance of helping to provide individuals with relevant information with appropriate language and terminology which can be used to describe CRCI to their friends, family, and workplace:

"I think really clear information for them…also really, really important to have appropriate terminology that they can take with them with when trying to describe it to their friends or their family or their school or workplace" (Lisa, Occupational Therapist).

Another health professional recognised the need for cancer survivors to be provided with additional information and resources about CRCI: *"the best to do it…it comes with a pamphlet that says, you know, a bit more information, this is resources available" (Marlene, Nurse)* as well as for the carers and loved ones at appointments:

"This other information is for you and then it can also be used to support the carers or support people who may or may not be at the appointment…even the children and extended family" (Marlene, Nurse).

As pointed out by a health professional, providing appropriate information about CRCI may be protective of mental health difficulties that manifest because of the confusion and distress associated with a lack of understanding of CRCI:

"I think that, you know, mental health support. Think pre-emptively, actually letting people know what they can expect is helpful because they then they know…then its yes, I was told about this" (Robin, Counsellor).
